# Automatic segmentation of the urethra and prostate zones with deep learning on T2-weighted magnetic resonance imaging

**DOI:** 10.1016/j.phro.2026.100964

**Published:** 2026-04-07

**Authors:** William Holmlund, Attila Simkó, Karin Söderkvist, Péter Palásti, Szilvia Tótin, Kamilla Kalmár, Zsófia Domoki, Zsuzsanna Fejes, Tamás Z. Kincses, Patrik Brynolfsson, Tufve Nyholm

**Affiliations:** a Umeå University, Department of Diagnostics and Intervention, Umeå, Sweden; b University of Szeged, Albert Szent-Györgyi Medical School, Department of Radiology, Szeged, Hungary; c Skåne University Hospital, Department of Haematology, Oncology and Radiation Physics, Lund, Sweden

**Keywords:** Automatic Segmentation, Urethra, Prostate Zones

## Abstract

**Background and purpose:**

Accurate segmentation of the urethra is crucial for safe focal dose escalated radiotherapy, while prostate zone identification is important for prostate cancer diagnosis. Manual delineations on magnetic resonance imaging (MRI) are labour-intensive and variable, and while deep learning offers promise in automating this process, no available solution currently exists. This study aimed to develop and evaluate a deep learning model for automatic segmentation of the urethra, prostate and all prostate zones and benchmark its performance against inter-reader variability and assess generalisability to external data from a different MRI vendor.

**Materials and methods:**

The public datasets ProstateZones and PROSTATEx included 200 magnetic resonance images with manual delineations, with 160 used for training/validation and 40 with independent duplicate segmentations used as a test set. A nnU-Net deep learning model was evaluated on the unseen test set and externally validated on a dataset with 55 samples. Performance was assessed using Dice Similarity Coefficient (DSC), Surface DSC, percentile Symmetric Surface Distance, and Center Line Distance (CLD) metrics.

**Results:**

The model outperformed the inter-reader variability on multiple structures, and notably on all metrics for the urethra, with median CLD values of 2.8 and 2.9 mm compared to 3.6 mm for inter-reader variability. External validation showed robust generalisability to a dataset collected from a different vendor.

**Conclusions:**

This study demonstrated that a deep learning model can achieve expert-level performance in automated segmentation of the urethra, prostate, and prostate zones. Robust performance on external data highlighted potential as a decision support solution.

## Highlights

• Deep learning based automated segmentation of urethra and prostate zones.
• Model segmented the urethra with a median Center Line Distance of 2.8–2.9 mm.
• Urethra segmentation was closer to each reader than the readers were to each other.
• Model showed generalisability across data from different vendors.
• Training and test data, model weights, and code repositories are publicly available.

## Graphical abstract

**Figure:**
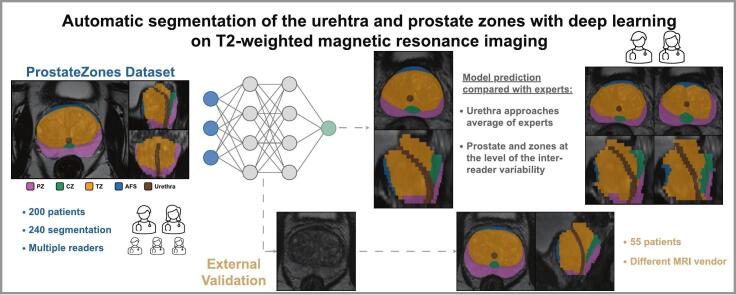

## 1 Introduction

Prostate cancer (PCa) is the most prevalent malignancy in males worldwide [1], requiring accurate diagnostic and treatment approaches. Magnetic resonance imaging (MRI) is recommended for diagnosis of clinically significant PCa [2], with the global prostate imaging reporting and data system (PI-RADS) guidelines aiming to improve standardisation and interpretation [3]. The guidelines specify that the assessment of prostatic lesions on multiparametric MRI (mpMRI) should use different primary sequences based on their zonal location. These anatomical zones of the prostate are defined as the peripheral zone (PZ), central zone (CZ), transitional zone (TZ), and anterior fibromuscular stroma (AFS), each presenting different characteristics and histological features [4]. Additionally, the guidelines recommend reporting prostate specific antigen density (PSAd), which has been linked to the presence of clinically significant PCa [5]. Further, TZ-specific PSAd has been reported to improve discrimination of clinically significant PCa and reduce false-positive MRI findings [6]. Moreover, accurate identification of lesion location is important for targeted biopsies, which has been shown to reduce the number of unnecessary biopsies and reduce diagnosis of clinically insignificant cancers [7], [8].

Traditionally, radiotherapy of PCa has been delivered with a homogeneous dose to the entire prostate, although the efficacy of local dose escalation to a sub volume of the prostate has been demonstrated [9]. The risk of an increased dose to the prostatic urethra is a clinical concern when escalating the radiotherapy dose inside the prostate. A combined analysis by Leeman et al. [10] demonstrated a significant association between urethral dose and genitourinary (GU) toxicity, including acute grade ≥2 and late ≥3 GU toxicity. The risk for urethral side effects is mitigated by using dose constraints, hence requiring delineation of the urethral structure [11]. Moreover, the effects of a local dose escalation could potentially be enhanced by utilising zonal information to individualise treatment and stratify risk, as location of the cancer in different zones have different incidence, prognosis, and outcome, making treatment zonal-dependent instead of zonal-agnostic [12].

Manual delineations of these structures on MRI are tedious and time-consuming. Moreover, they are subject to inter- and intra-reader variability due to low image contrast and patient-specific differences in prostate anatomy [13]. Deep learning models have been well-documented in addressing challenges in medical image analysis overall [14], [15], and particularly for segmentations of prostate MRI [16], [17].

Despite the emerging clinical importance of accurately delineating the prostatic urethra, only a limited number of studies have been published. Multiple studies used models that relied on manual delineations with a Foley catheter in place [18], [19], [20], [21]. However, the catheter impacts the anatomical configuration of the prostate and urethra, which makes the results unreliable for a non-catheter scenario [22], [23]. MRI-based approaches offer a non-invasive alternative, leveraging its superior soft tissue contrast. Nonetheless, only a few studies have explored automatic segmentation of the urethra on MRI [24], [25], [26], [27]. For all these studies, it is important to note that their methods were developed using proprietary datasets, and one study optimised image sequences for visualisation of the urethra [26]. Furthermore, all delineations were carried out by supervised medical students [25] or a single expert [24], [26], [27], and none of the models have been made publicly available, with only one using an external test set [27].

For the prostate zones, previous studies have investigated multiple variants of the U-Net architecture for segmentation of the prostate gland and zonal structures, with most differentiating only between PZ and non-PZ regions [28], [29], [30], [31], [32], [33], [34], [35], [36], [37], [38]. For example, Aldoj et al. [28] developed a Dense U-Net for automatic segmentation of the prostate, PZ and CZ. Bardis et al. [29] utilised a combination of three customised hybrid 3D/2D U-Nets to localise and segment the prostate and subsequently classify the PZ and TZ. Xu et al. [35] used a 3D U-Net model to segment the PZ and central gland (CG). Jimenez-Pastor et al. [33] employed a U-Net with deep supervision to automatically segment the prostate, PZ, a combined CZ-TZ, and seminal vesicles. All these studies segmented the prostate into sub-regions, described as PZ and non-PZ regions, with a clear inconsistency in terminology. This varying terminology and definition of delineated structures in the existing literature is a major limitation for clinical applicability, as was highlighted in a topical review [39]. Only a study by Meyer et al. [38], which delineated the PZ, CG (a combined delineation of CZ and TZ), AFS, and the distal prostatic urethra, included more than two intra-prostatic structures. Furthermore, the topical review highlighted a general lack of inter-reader variability data and that a vast majority of data were private, making comparisons of the performance between models difficult.

This study aimed to implement a deep learning model for automatic segmentation of the complete intra-prostatic urethra, prostate, and all four prostate zones, using structure terminology that adheres to the PI-RADS guidelines. The model was evaluated relative to inter-reader variability between a pair of radiologists, and its generalisability was assessed on an external dataset acquired from a different MRI vendor. Additionally, this work established a benchmark for a publicly available segmentation dataset with open code repositories to enable reproducible and continued research.

## 2 Materials and methods

### 2.1 Data

In this work, the publicly available PROSTATEx [40], [41] dataset was used, along with our previously published ProstateZones [42], [43] segmentation dataset, which included segmentations for 200 randomly selected patients from PROSTATEx. The segmentations were manually delineated pixel-wise on axial T2w MRIs by two experienced radiologists (P.P., Z.F.) in collaboration with three junior colleagues (K.K., S.T., Z.D.). Prostate zones, including the PZ, CZ, TZ, and AFS, were delineated, and the urethra was defined as a six mm diameter circular region in each slice where present [43].

Out of the 200 segmentations, 160 samples had one segmentation per structure and were used for training and validating the deep learning model, while 40 samples had two segmentations per structure and were used as a test set. The duplicate delineations were performed independently by two experienced radiologists, which provided an inter-reader variability baseline for assessing model performance.

### 2.2 Deep learning model

A nnU-Net (v.2.2) [44] was trained on an NVIDIA GeForce RTX 3090 GPU with five-fold cross-validation on the 160 training samples of the dataset. The model performed semantic segmentations across all six classes (background, urethra and four prostate zones) using the image volumes. Code, weights and training details of the model are available with further details (https://github.com/UMU-DDI/prostate-segmentation).

### 2.3 Post-processing

Post-processing was used to align the model output with the manual delineation structure of the urethra, represented as a single circle in each slice with a fixed diameter of six mm.

First, the voxel-wise probability maps were used to extract the prostate volume as the largest connected component from the inverse of the background. Second, the coordinates of the voxel with the highest probability of being the urethra was extracted for each slice. If multiple voxels had the same probability, the centre of mass of the voxels was used. Third, a three-dimensional 2^nd^ degree polynomial was fitted to the coordinates, with the initial probabilities used as weights. The polynomial fit output was used as the centre around which a new urethra was drawn with the predetermined circular shape and diameter of six mm, restricted by the prostate volume. Then, the largest connected components for each of the prostate zones were determined. Finally, any remaining voxels within the predicted prostate volume were assigned based on minimum Euclidean distance to the zones, emphasising anatomical correctness. This fully automated post-processing setup created a structure priority scheme, which emphasised the prostate boundary and urethra.

Example predictions with and without post-processing are displayed in supplementary Figs. S1-S2.

### 2.4 Evaluation metrics

The performance of the model was evaluated with the Dice Similarity Coefficient (DSC), Surface DSC (SDSC) [45], percentile Symmetric Surface Distance (pSSD), and Center Line Distance (CLD). DSC quantified volumetric overlap, while SDSC captured boundary agreement within a specified tolerance to reflect typical inter-reader variability. The pSSD provided the symmetric surface distance required to include a given percentile of all boundary points, offering a tolerance-free, distribution-based measure directly related to the SDSC. Finally, the CLD evaluated the average symmetric distance between two skeletonised versions of the urethra.

Difference between model and reader variation was tested for statistical significance using the related-samples Wilcoxon Signed Rank Test, as the data was determined to be non-Gaussian distributed based on the Shapiro-Wilk test. The same non-parametric test was also applied to quantify the effect of the post-processing on segmentation performance. Dataset differences in prostate volume and zonal proportions were tested using Welch’s t-test on transformed data, while associations between CLD and anatomical structures were examined using correlation and linear regression on log10-transformed volumes. No correction for multiple testing was performed. All evaluations were performed in Hero v.2025.2 (Hero Imaging AB, Umeå, Sweden; https://www.heroimaging.com/) with subsequent statistical analysis performed in SPSS Statistics v.29.0.1.0 (IBM, Armonk, NY, USA).

### 2.5 External test set

To assess the generalisability of the model, external validation was performed on a private dataset, which consisted of a prospective collection of 55 intermediate and high-risk prostate cancer patients from an ethically approved in-house study at the University Hospital of Umeå (Regional Ethics Board approval: Dnr 2016-220-31 M) [46].

Segmentations of the urethra and prostate zones were delineated slice-wise on the axial T2w MRI in the clinical software RayStation v.2023B (RaySearch Laboratories, Stockholm, Sweden). They followed the same delineation procedure as the ProstateZones dataset, including a fixed 6 mm circular urethra, and were performed by a medical physicist (W.H.) and subsequently controlled and corrected by an experienced radiation oncologist (K.S.). Images were collected with a Signa PET/MR 3 T scanner (GE Healthcare, Chicago, IL, USA) and had a reconstructed in-plane resolution of 0.41 mm and a slice thickness of 2.5 mm.

The pre- and post-processing of the external data was performed in the same manner as the other test data.

## 3 Results

**Fig. 1:**
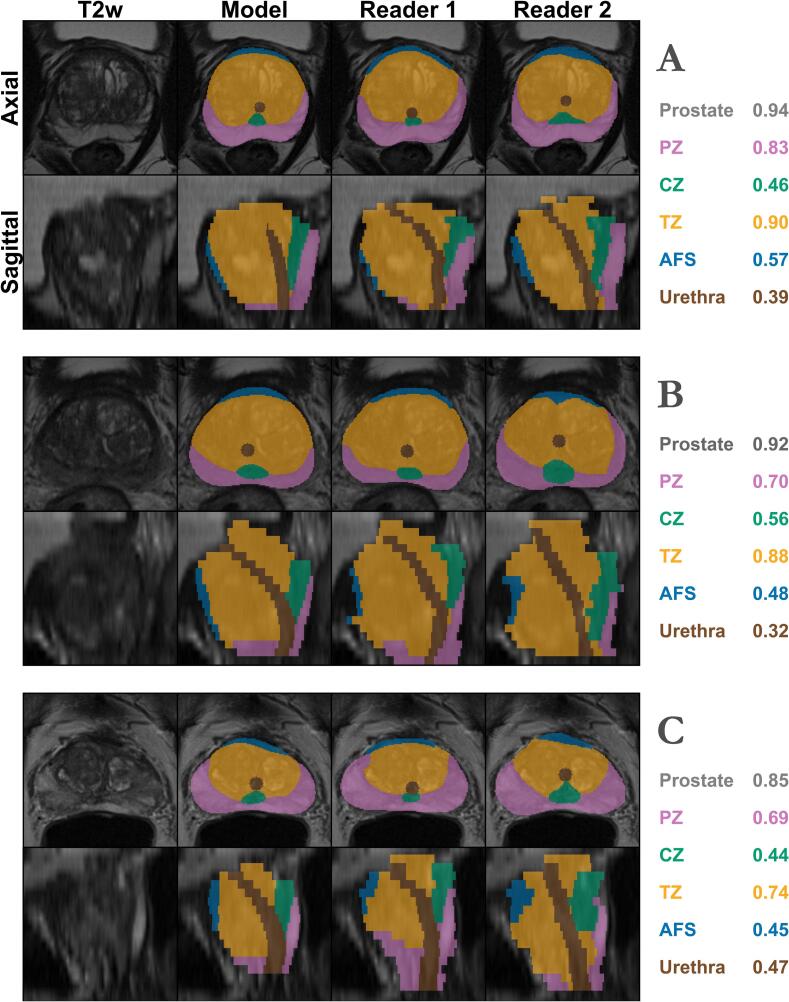
Example segmentations on the test data. Examples with the smallest (panel A), average (panel B), and largest (panel C) differences in DSC between manual and automatic segmentations*.* The average model DSC for each structure is presented for each case to the right in each panel. (For interpretation of the references to colour in this figure legend, the reader is referred to the web version of this article.)

Inter-reader variability on the test set showed a median CLD of 3.6 mm, with all metrics summarized in Table 1. Model comparisons showed a median CLD of 2.9 and 2.8 mm compared to Reader 1 and 2, respectively, as detailed in Table 2, with representative examples displayed in Fig. 1.

**Table 1:** Inter-reader variability for the test data (n = 40). Metrics are presented as the median and inter-quartile ranges.

	**DSC**	**SDSC**	**pSSD *(mm)***			**CLD *(mm)***
		*1 mm*	*P_50_*	*P_80_*	*P_95_*	
**Prostate**	0.92 [0.90, 0.93]	0.71 [0.66, 0.73]	0.50 [0.50, 0.52]	1.5 [1.4, 1.8]	3.0 [3.0, 3.0]	−
**PZ**	0.75 [0.72, 0.80]	0.67 [0.65, 0.71]	0.50 [0.50, 0.56]	1.8 [1.6, 2.1]	3.7 [3.2, 4.7]	−
**CZ**	0.44 [0.35, 0.56]	0.41 [0.30, 0.50]	2.15 [1.12, 3.00]	6.0 [3.4, 7.5]	10.4 [7.7, 12.4]	−
**TZ**	0.84 [0.79, 0.87]	0.59 [0.56, 0.62]	0.71 [0.56, 1.00]	2.4 [2.1, 2.7]	3.6 [3.3, 3.9]	−
**AFS**	0.39 [0.26, 0.48]	0.52 [0.44, 0.57]	1.00 [0.71, 1.52]	3.2 [3.0, 4.2]	6.1 [4.5, 8.9]	−
**Urethra**	0.33 [0.25, 0.43]	0.42 [0.33, 0.49]	1.54 [1.12, 2.50]	3.9 [2.9, 4.8]	6.2 [4.1, 7.2]	3.6 [2.8, 4.4]
DSC: Dice Similarity Coefficient, SDSC: Surface DSC, pSSD: Percentile Symmetric Surface Distance, CLD: Center Line Distance, PZ: Peripheral zone, CZ: Central zone, TZ: Transitional zone, AFS: Anterior fibromuscular stroma.

**Table 2:** Performance metrics for the model stratified for both readers, with comparisons towards Reader 1 presented in the upper half and Reader 2 in the lower half. Metrics are presented as the median and inter-quartile ranges.

*Model*		**DSC**	**SDSC**	**pSSD *(mm)***			**CLD *(mm)***
			*1 mm*	*P_50_*	*P_80_*	*P_95_*	
Reader 1	**Prostate**	0.91 [0.90, 0.92]	0.69 [0.63, 0.73]	0.50 [0.50, 0.56]	1.6 [1.5, 2.1]	3.2 * [3.0, 3.6]	−
	**PZ**	0.78 [0.72, 0.80]	0.70 [0.63, 0.74]	0.50 [0.50, 0.52]	1.7 [1.4, 2.1]	3.5 [3.0, 4.4]	−
	**CZ**	0.50 * [0.43, 0.56]	0.47 * [0.40, 0.54]	1.31 * [1.00, 1.58]	4.7 [3.9, 6.1]	9.5 [8.4, 11.1]	−
	**TZ**	0.86 * [0.83, 0.88]	0.61 * [0.57, 0.65]	0.64 [0.50, 0.85]	2.1 * [1.8, 2.4]	3.2 * [3.0, 3.6]	−
	**AFS**	0.42 [0.34, 0.52]	0.59 * [0.49, 0.67]	0.71 [0.50, 1.12]	3.0 * [1.9, 3.4]	4.5 * [3.4, 7.1]	−
	**Urethra**	0.41 * [0.36, 0.52]	0.49 * [0.41, 0.56]	1.10 * [0.71, 1.41]	3.0 * [2.7, 3.5]	4.0 * [3.5, 5.5]	2.9 * [2.3, 3.2]
Reader 2	**Prostate**	0.92 [0.91, 0.93]	0.72 [0.67, 0.76]	0.50 [0.50, 0.52]	1.5 [1.2, 1.8]	3.0 [3.0, 3.2]	−
	**PZ**	0.75 [0.73, 0.80]	0.71 * [0.66, 0.74]	0.50 [0.50, 0.52]	1.6 [1.4, 2.1]	3.5 [3.0, 5.1]	−
	**CZ**	0.54 * [0.45, 0.61]	0.49 * [0.42, 0.59]	1.12 * [0.68, 1.50]	3.0 * [2.7, 3.8]	5.8 * [4.3, 7.8]	−
	**TZ**	0.85 * [0.82, 0.87]	0.59 * [0.57, 0.64]	0.56 * [0.50, 0.71]	2.1 * [2.0, 2.5]	3.4 * [3.1, 3.7]	−
	**AFS**	0.53 * [0.40, 0.58]	0.60 * [0.46, 0.64]	0.71 [0.50, 1.41]	3.0 * [2.4, 3.2]	5.0 [3.6, 6.8]	−
	**Urethra**	0.41 * [0.30, 0.52]	0.49 * [0.39, 0.62]	1.12 * [0.67, 1.80]	3.0 * [2.2, 3.6]	4.0 * [3.2, 5.1]	2.8 * [2.3, 3.7]
	DSC: Dice Similarity Coefficient, SDSC: Surface DSC, pSSD: Percentile Symmetric Surface Distance, CLD: Center Line Distance, PZ: Peripheral zone, CZ: Central zone, TZ: Transitional zone, AFS: Anterior fibromuscular stroma. *: Statistically significant difference relative to the inter-reader variability (p < 0.05).

Prediction and post-processing of all 40 cases required less than 10 minutes, for an average of 14.7 seconds per sample.

All statistical differences indicated lower model-reader variation compared to the inter-reader variation, except for the 95^th^ pSSD for the prostate compared to Reader 1 (Table 2). Compared to the initial prediction, the applied post-processing mainly influenced the urethra, with a mean improvement for the CLD of -0.71 mm, as shown in supplementary Table S1.

**Fig. 2:**
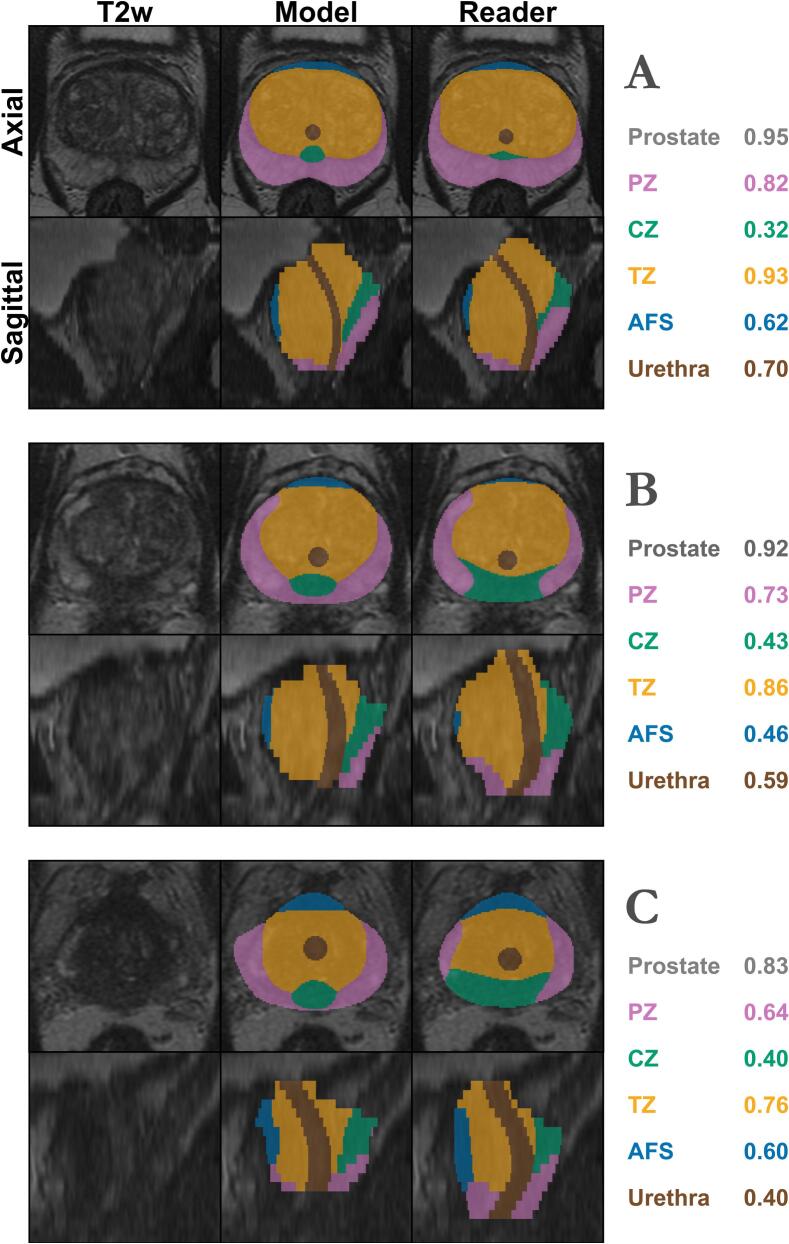
Example segmentations on the external validation data. Examples with the smallest (panel A), average (panel B), and largest (panel C) differences in DSC between manual and automatic segmentations. The model DSC for each structure is presented for each case to the right in each panel. (For interpretation of the references to colour in this figure legend, the reader is referred to the web version of this article.)

On the external test set, the model achieved a median CLD of 2.3 mm, demonstrating robust generalisability as summarized in Table 3, with representative examples displayed in Fig. 2.

**Table 3:** Performance metrics for the model on the external test set (n = 55). Metrics are presented as the median and inter-quartile ranges.

	**DSC**	**SDSC**	**pSSD *(mm)***			**CLD *(mm)***
		*1 mm*	*P_50_*	*P_80_*	*P_95_*	
**Prostate**	0.91 [0.89, 0.92]	0.74 [0.71, 0.78]	0.41 [0.41, 0.41]	1.5 [1.2, 1.7]	2.9 [2.5, 3.6]	−
**PZ**	0.80 [0.76, 0.83]	0.75 [0.70, 0.78]	0.41 [0.41, 0.41]	1.2 [1.2, 1.9]	2.8 [2.5, 3.7]	−
**CZ**	0.41 [0.35, 0.46]	0.35 [0.27, 0.41]	2.32 [1.72, 2.58]	6.5 [5.2, 7.7]	11.1 [9.6, 12.6]	−
**TZ**	0.81 [0.79, 0.86]	0.64 [0.59, 0.69]	0.58 [0.41, 0.82]	2.1 [1.7, 2.2]	3.5 [2.9, 4.8]	−
**AFS**	0.56 [0.45, 0.61]	0.69 [0.59, 0.77]	0.41 [0.41, 0.82]	1.6 [1.2, 2.5]	3.3 [2.7, 5.0]	−
**Urethra**	0.49 [0.37, 0.68]	0.53 [0.42, 0.73]	0.92 [0.58, 1.30]	2.5 [1.3, 3.0]	3.7 [2.7, 5.0]	2.3 [1.5, 3.2]
DSC: Dice Similarity Coefficient, SDSC: Surface DSC, pSSD: Percentile Symmetric Surface Distance, CLD: Center Line Distance, PZ: Peripheral zone, CZ: Central zone, TZ: Transitional zone, AFS: Anterior fibromuscular stroma.

For the external dataset, prediction and post-processing of all 55 cases required just over 15 minutes, for an average of 16.6 seconds per sample on the same system as used during training.

Systematic anatomical differences were observed between the test set and the external validation dataset, as detailed in supplementary Tables S2–S3. The test set exhibited significantly larger prostate volumes (mean 58.5 cm^3^ vs. 40.8 cm^3^; p<0.001) and altered zonal composition, including a lower proportion of CZ (Odds Ratio (OR) = 0.48; 95% CI: 0.40-0.58; p<0.001) and a higher proportion of TZ (OR = 1.45; 95% CI: 1.16-1.81; p=0.001). Additionally, model performance for the urethra segmentation showed correlation with anatomical factors, with the strongest association for the CLD observed with increased TZ volume for both datasets (Pearson r=0.54-0.56), as detailed in supplementary Table S4.

## 4 Discussion

In this study, we implemented a deep learning model for the segmentation of the urethra, prostate, and prostate zones, with evaluation on unseen test data containing segmentations from multiple readers and an external dataset to assess generalisability. Overall, performance on the test set was at the level of, or better than, the inter-reader variability, with robust predictions on the external test set. Notably, when compared to the inter-reader variability, urethra segmentations were significantly closer to each radiologist than the radiologists were to each other, indicating that the model output tended towards an average of both on unseen test data.

In the existing literature, the urethra segmentation of Czipczer et al. [25], achieved a DSC of 0.39 and a 1 mm SDSC of 0.43 when segmenting the urethra as an organ at risk for radiotherapy planning. In a study with over 900 total patients, Belue et al. [24] achieved a DSC of 0.59 and a CLD of 2.56 mm, although their CLD was computed slice-wise and only included slices with both segmentations present, meaning that any slice with only one segmentation was not penalised. Furthermore, all segmentations were performed by a single delineator, a known limitation due to potential bias introduced in predictions when compared to the same delineator [38]. In contrast, our evaluations included all slices, meaning that any slice with only one segmentation would register a distance of at least the slice thickness. Additionally, a strength of our study was that it involved multiple delineators in the delineation process for the training data, and that evaluations were performed against three different delineators in total. Xu et al. [26] reported that manual delineations of the urethra typically required 5 minutes per sample, which is substantially longer than our complete pipeline, which processed each sample in, on average less than 17 seconds in both the test and external validation datasets.

Comparing segmentations of prostate zones is challenging due to varying terminology and structure definitions used in previous studies. However, the literature reports mean or median DSC values for the prostate, PZ, and TZ/CZ/CZ-TZ/CG in the ranges of 0.84-0.95, 0.70-0.87, and 0.85-0.94, respectively [25], [28], [29], [30], [31], [32], [33], [34], [35], [36], [37], [38]. A single study that delineated the AFS reported a DSC of 0.41 [38]. This was in line with our results; however, differentiating the prostate into a larger number of structures is inherently more challenging. Similarly, as far as a comparison is possible, the inter-reader variability, presented in Table 1, was comparable with previous studies [13], [28], [31], [47], [48].

This study included limitations. First, the model used did not reflect the contemporary state-of-the-art, such as transformer-inspired models like MedNeXt [49]. However, rather than optimising for state-of-the-art performance, our primary goal was to establish a publicly accessible and reproducible benchmark for segmentation of the urethra and prostate zones. The focus was therefore on evaluation against inter-reader variability and external validation across vendors, and open access to data and code, rather than model innovation. Still, the fact that a lightweight model like the nnU-Net could reach segmentation performance at the level of the inter-reader variability demonstrated that this task can be achieved without requiring large computational resources.

Second, the resolution of the images from PROSTATEx did not comply with the recommendations of the PI-RADS v2.1 guidelines, where adherence has been shown to make models more generalisable [50]. Additionally, training data consisted solely of images from a single vendor (Siemens), which has been shown to degrade performance when evaluated on data from a different vendor (GE), compared to models trained on data from multiple vendors [37]. Although we did not compare our model with one trained on data from multiple vendors, our results were somewhat contrary to these findings. Several factors may have contributed to this outcome. For example, the external data had higher resolution, which likely supported clearer depiction of both zonal boundaries and the urethra. Additionally, the datasets differed anatomically, including variations in prostate size and zonal proportions. These anatomical differences may partly reflect variation in reader background and experience, particularly in how anatomical boundaries were interpreted. In our analysis, larger prostates and, in particular, a larger TZ were associated with greater urethral variability, reflected by increased CLD values. Enlarged TZ volumes are often linked to benign prostatic hyperplasia, a condition known to distort gland morphology and complicate delineations of the urethra [3], [24]. Notably, both total prostate and TZ volume were larger in the test set than in the external data, which may partially explain why the deviation in urethral segmentations were more pronounced there.

Third, the urethral ground truth relied on a heuristic delineation protocol, in which the centre of the urethra was identified on each slice and represented using a fixed 6 mm circular contour, with interpolation applied in low-visibility slices. While this approach is clinically practical and consistent across datasets, it is not an accurate representation of the true urethral anatomy, something that should be considered in future studies. However, as the post-processing worked under the same heuristics, comparison within the study remained valid.

Fourth, while this study included post-processing that emphasised the urethra and prostate boundary for its clear importance in local dose escalated radiotherapy, it did not evaluate the clinical applicability of these segmentations within a radiotherapy workflow.

In conclusion, this study implemented a deep learning model, the nnU-Net, for segmentations of the urethra, prostate, and prostate zones with performance comparable to the inter-reader variability of two radiologists on unseen test data. Notably, the model provided results significantly better than the inter-reader variability for multiple structures and metrics, especially for the urethra, with only a single comparison in total being significantly worse. The segmentation of the urethra approached an average of the manual delineations, as the output was on average closer to each delineator than they were to each other. Additionally, performance was robust when generalisability was assessed on external test data collected from a different vendor. These findings showed the potential to reduce time and variability associated with manual delineations of the urethra, prostate, and prostate zones. By achieving segmentation quality comparable to manual delineations in seconds instead of minutes, this approach showed promise as a decision support solution, although further validation is needed in clinical settings. Furthermore, this work addressed an important gap in the field by presenting a fully automatic solution that theoretically impacts both diagnostic and therapeutic applications. Finally, it established a benchmark for a publicly available segmentation dataset with open code repositories to enable reproducible and continued research.

## CRediT authorship contribution statement

**William Holmlund:** Writing – review & editing, Writing – original draft, Methodology, Data curation, Conceptualization. **Attila Simkó:** Writing – review & editing, Methodology, Conceptualization. **Karin Söderkvist:** Writing – review & editing, Data curation, Conceptualization. **Péter Palásti:** Writing – review & editing, Data curation. **Szilvia Tótin:** Writing – review & editing, Data curation. **Kamilla Kalmár:** Writing – review & editing, Data curation. **Zsófia Domoki:** Writing – review & editing, Data curation. **Zsuzsanna Fejes:** Writing – review & editing, Data curation. **Tamás Z. Kincses:** Supervision and Writing – review & editing. **Patrik Brynolfsson:** Writing – review & editing, Conceptualization. **Tufve Nyholm:** Writing – review & editing, Supervision, Conceptualization.

## Declaration of competing interest

The authors declare the following financial interests/personal relationships which may be considered as potential competing interests: TN, PB and AS are co-owners of Hero Imaging AB, developing the software used during evaluations. All other authors declare no conflict of interest.

## References

[1] International Agency for Research on Cancer. Global Cancer Observatory: Cancer Today, https://gco.iarc.who.int/today/en ; 2024 [accessed 10 February 2026].

[2] Mottet N., van den Bergh R.C.N., Briers E., Van den Broeck T., Cumberbatch M.G., De Santis M. (2021). EAU-EANM-ESTRO-ESUR-SIOG Guidelines on Prostate Cancer—2020 Update. Part 1: Screening, Diagnosis, and Local Treatment with Curative Intent. Eur Urol.

[3] Turkbey B., Rosenkrantz A.B., Haider M.A., Padhani A.R., Villeirs G., Macura K.J. (2019). update of Prostate Imaging Reporting and Data System version 2. Eur Urol.

[4] McNeal J.E. (1988). Normal histology of the prostate. Am J Surg Pathol..

[5] Haj-Mirzaian A., Burk K.S., Lacson R., Glazer D.I., Saini S., Kibel A.S. (2024). Magnetic Resonance Imaging, Clinical, and Biopsy Findings in Suspected Prostate Cancer. A Systematic Review and Meta-Analysis. JAMA Netw Open..

[6] Hamm C.A., Baumgärtner G.L., Padhani A.R., Froböse K.P., Dräger F., Beetz N.L. (2024). Reduction of false positives using zone-specific prostate-specific antigen density for prostate MRI-based biopsy decision strategies. Eur Radiol..

[7] Kasivisvanathan V., Rannikko A.S., Borghi M., Panebianco V., Mynderse L.A., Vaarala M.H. (2018). MRI-Targeted or Standard Biopsy for Prostate-Cancer Diagnosis. N Engl J Med..

[8] Eklund M., Jäderling F., Discacciati A., Bergman M., Annerstedt M., Aly M. (2021). MRI-Targeted or Standard Biopsy in Prostate Cancer Screening. N Engl J Med..

[9] Kerkmeijer L.G.W., Groen V.H., Pos F.J., Haustermans K., Monninkhof E.M., Smeenk R.J. (2021). Focal Boost to the Intraprostatic Tumor in External Beam Radiotherapy for Patients With Localized Prostate Cancer: Results From the FLAME Randomized Phase III Trial. J Clin Oncol..

[10] Leeman J.E., Chen Y.H., Catalano P., Bredfeldt J., King M., Mouw K.W. (2022). Radiation Dose to the Intraprostatic Urethra Correlates Strongly With Urinary Toxicity After Prostate Stereotactic Body Radiation Therapy: A Combined Analysis of 23 Prospective Clinical Trials. Int J Radiat Oncol Biol Phys..

[11] Groen V.H., van Schie M., Zuithoff N.P.A., Monninkhof E.M., Kunze-Busch M., de Boer J.C.J. (2022). Urethral and bladder dose-effect relations for late genitourinary toxicity following external beam radiotherapy for prostate cancer in the FLAME trial. Radiother Oncol..

[12] Ali A., Du Feu A., Oliveira P., Choudhury A., Bristow R.G., Baena E. (2022). Prostate zones and cancer: lost in transition?. Nat Rev Urol..

[13] Montagne S., Hamzaoui D., Allera A., Ezziane M., Luzurier A., Quint R. (2021). Challenge of prostate MRI segmentation on T2-weighted images: inter-observer variability and impact of prostate morphology. Insights Imaging..

[14] Litjens G., Kooi T., Bejnordi B.E., Setio A.A.A., Ciompi F., Ghafoorian M. (2017). A survey on deep learning in medical image analysis. Med Image Anal..

[15] Wang R., Lei T., Cui R., Zhang B., Meng H., Nandi A.K. (2022). Medical image segmentation using deep learning: A survey. IET Image Process..

[16] Turkbey B., Haider M.A. (2022). Deep learning-based artificial intelligence applications in prostate MRI: brief summary. Br J Radiol..

[17] Khan Z., Yahya N., Alsaih K., Al-Hiyali M.I., Meriaudeau F. (2021). Recent Automatic Segmentation Algorithms of MRI Prostate Regions: A Review. IEEE Access..

[18] Elguindi S., Zelefsky M.J., Jiang J., Veeraraghavan H., Deasy J.O., Hunt M.A. (2019). Deep learning-based auto-segmentation of targets and organs-at-risk for magnetic resonance imaging only planning of prostate radiotherapy. Phys Imaging Radiat Oncol..

[19] Acosta O., Mylona E., Le Dain M., Voisin C., Lizee T., Rigaud B. (2017). Multi-atlas-based segmentation of prostatic urethra from planning CT imaging to quantify dose distribution in prostate cancer radiotherapy. Radiother Oncol..

[20] Takagi H., Kadoya N., Kajikawa T., Tanaka S., Takayama Y., Chiba T. (2020). Multi-atlas-based auto-segmentation for prostatic urethra using novel prediction of deformable image registration accuracy. Med Phys..

[21] Cubero L., Garcia-Elcano L., Mylona E., Boue-Rafle A., Cozzarini C., Ubeira Gabellini M.G. (2023). Deep learning-based segmentation of prostatic urethra on computed tomography scans for treatment planning. Phys Imaging Radiat Oncol..

[22] Litzenberg D.W., Muenz D.G., Archer P.G., Jackson W.C., Hamstra D.A., Hearn J.W. (2018). Changes in prostate orientation due to removal of a Foley catheter. Med Phys..

[23] Dekura Y., Nishioka K., Hashimoto T., Miyamoto N., Suzuki R., Yoshimura T. (2019). The urethral position may shift due to urethral catheter placement in the treatment planning for prostate radiation therapy. Radiat Oncol..

[24] Belue M.J., Harmon S.A., Patel K., Daryanani A., Yilmaz E.C., Pinto P.A. (2022). Development of a 3D CNN-based AI Model for Automated Segmentation of the Prostatic Urethra. Acad Radiol..

[25] Czipczer V., Kolozsvári B., Deák-Karancsi B., Capala M.E., Pearson R.A., Borzási E. (2023). Comprehensive deep learning-based framework for automatic organs-at-risk segmentation in head-and-neck and pelvis for MR-guided radiation therapy planning. Front Phys..

[26] Xu D., Ma T.M., Savjani R., Pham J., Cao M., Yang Y. (2023). Fully automated segmentation of prostatic urethra for MR-guided radiation therapy. Med Phys..

[27] Song Y., Nguyen L., Dornisch A.M., Baxter M.T., Barrett T., Dale A.M. (2026). Urethra contours on MRI: Multidisciplinary consensus educational atlas and reference standard for artificial intelligence benchmarking. Radiother Oncol.

[28] Aldoj N., Biavati F., Michallek F., Stober S., Dewey M. (2020). Automatic prostate and prostate zones segmentation of magnetic resonance images using DenseNet-like U-net. Sci Rep..

[29] Bardis M., Houshyar R., Chantaduly C., Tran-Harding K., Ushinsky A., Chahine C. (2021). Segmentation of the Prostate Transition Zone and Peripheral Zone on MR Images with Deep Learning. Radiol Imaging Cancer..

[30] Cuocolo R., Comelli A., Stefano A., Benfante V., Dahiya N., Stanzione A. (2021). Deep Learning Whole-Gland and Zonal Prostate Segmentation on a Public MRI Dataset. J Magn Reson Imaging..

[31] Adams L.C., Makowski M.R., Engel G., Rattunde M., Busch F., Asbach P. (2022). Prostate158 - An expert-annotated 3T MRI dataset and algorithm for prostate cancer detection. Comput Biol Med..

[32] Hamzaoui D., Montagne S., Renard-Penna R., Ayache N., Delingette H. (2022). Automatic zonal segmentation of the prostate from 2D and 3D T2-weighted MRI and evaluation for clinical use. J Med Imaging..

[33] Jimenez-Pastor A., Lopez-Gonzalez R., Fos-Guarinos B., Garcia-Castro F., Wittenberg M., Torregrosa-Andres A. (2023). Automated prostate multi-regional segmentation in magnetic resonance using fully convolutional neural networks. Eur Radiol..

[34] Rodrigues N.M., Almeida J.G., Verde A.S.C., Gaivao A.M., Bilreiro C., Santiago I. (2024). Analysis of domain shift in whole prostate gland, zonal and lesions segmentation and detection, using multicentric retrospective data. Comput Biol Med..

[35] Xu L., Zhang G., Zhang D., Zhang J., Zhang X., Bai X. (2023). Development and clinical utility analysis of a prostate zonal segmentation model on T2-weighted imaging: a multicenter study. Insights Imaging..

[36] Zabihollahy F., Schieda N., Krishna Jeyaraj S., Ukwatta E. (2019). Automated segmentation of prostate zonal anatomy on T2‐weighted (T2W) and apparent diffusion coefficient (ADC) map MR images using U‐Nets. Med Phys..

[37] Zavala-Romero O., Breto A.L., Xu I.R., Chang Y.C., Gautney N., Dal Pra A. (2020). Segmentation of prostate and prostate zones using deep learning: A multi-MRI vendor analysis. Strahlenther Onkol..

[38] Meyer A., Rakr M., Schindele D., Blaschke S., Schostak M., Fedorov A. (2019). 2019 IEEE 16th Int Symp Biomed Imaging (ISBI).

[39] Wu C., Montagne S., Hamzaoui D., Ayache N., Delingette H., Renard-Penna R. (2022). Automatic segmentation of prostate zonal anatomy on MRI: a systematic review of the literature. Insights Imaging..

[40] [40] Litjens G, Debats O, Barentsz J, Karssemeijer N, Huisman H. SPIE-AAPM PROSTATEx Challenge Data, The Cancer Imaging Archive, v2; 2017. https://doi.org/10.7937/K9TCIA.2017.MURS5CL.

[41] Clark K., Vendt B., Smith K., Freymann J., Kirby J., Koppel P. (2013). The Cancer Imaging Archive (TCIA): maintaining and operating a public information repository. J Digit Imaging..

[42] Holmlund W., Simkó A., Söderkvist K., Palásti P., Tótin S., Kalmár K. (2024). ProstateZones – Segmentations of the prostatic zones and urethra for the PROSTATEx dataset. Zenodo.

[43] Holmlund W., Simkó A., Söderkvist K., Palásti P., Tótin S., Kalmár K. (2024). ProstateZones – Segmentations of the prostatic zones and urethra for the PROSTATEx dataset. Sci Data..

[44] Isensee F., Jaeger P.F., Kohl S.A.A., Petersen J., Maier-Hein K.H. (2021). nnU-Net: a self-configuring method for deep learning-based biomedical image segmentation. Nat Methods..

[45] Nikolov S., Blackwell S., Zverovitch A., Mendes R., Livne M., De Fauw J. (2021). Clinically Applicable Segmentation of Head and Neck Anatomy for Radiotherapy: Deep Learning Algorithm Development and Validation Study. J Med Internet Res..

[46] Nilsson E., Sandgren K., Grefve J., Jonsson J., Axelsson J., Keeratijarut Lindberg A. (2023). The grade of individual prostate cancer lesions predicted by magnetic resonance imaging and positron emission tomography. Commun Med..

[47] Becker A.S., Chaitanya K., Schawkat K., Muehlematter U.J., Hötker A.M., Konukoglu E. (2019). Variability of manual segmentation of the prostate in axial T2-weighted MRI: A multi-reader study. Eur J Radiol..

[48] Padgett K.R., Swallen A., Pirozzi S., Piper J., Chinea F.M., Abramowitz M.C. (2019). Towards a universal MRI atlas of the prostate and prostate zones. Strahlenther Onkol..

[49] Roy, S., Koehler, G., Ulrich, C., Baumgartner, M., Petersen, J., Isensee, F., Jäger, P. F., & Maier‑Hein, K. H. MedNeXt: Transformer‑Driven Scaling of ConvNets for Medical Image Segmentation. In: Medical Image Computing and Computer‑Assisted Intervention – MICCAI 2023. Lecture Notes in Computer Science, vol. 14223. 2023:405–415. https://doi.org/10.1007/978-3-031-43901-8_39.

[50] Fernandez-Quilez A, Nordström T, Eftestøl T, Alvestad AB, Jäderling F, Kjosavik SR, et al. Revisiting prostate segmentation in magnetic resonance imaging (MRI): On model transferability, degradation and PI-RADS adherence. [preprint] medRxiv. 2023:2023.08.21.23294376. https://doi.org/10.1101/2023.08.21.23294376.

