# Supplementary material for: Automatic segmentation of the urethra and prostate zones with deep learning on T2-weighted magnetic resonance imaging
Source: Phys Imaging Radiat Oncol. 2026 Apr 7;38:100964. doi: 10.1016/j.phro.2026.100964 (PMC13091743; doi:10.1016/j.phro.2026.100964)
Supplement: Supplementary Data 1 [file mmc1.pdf]

Supplementary Material

Analysis of post-processing

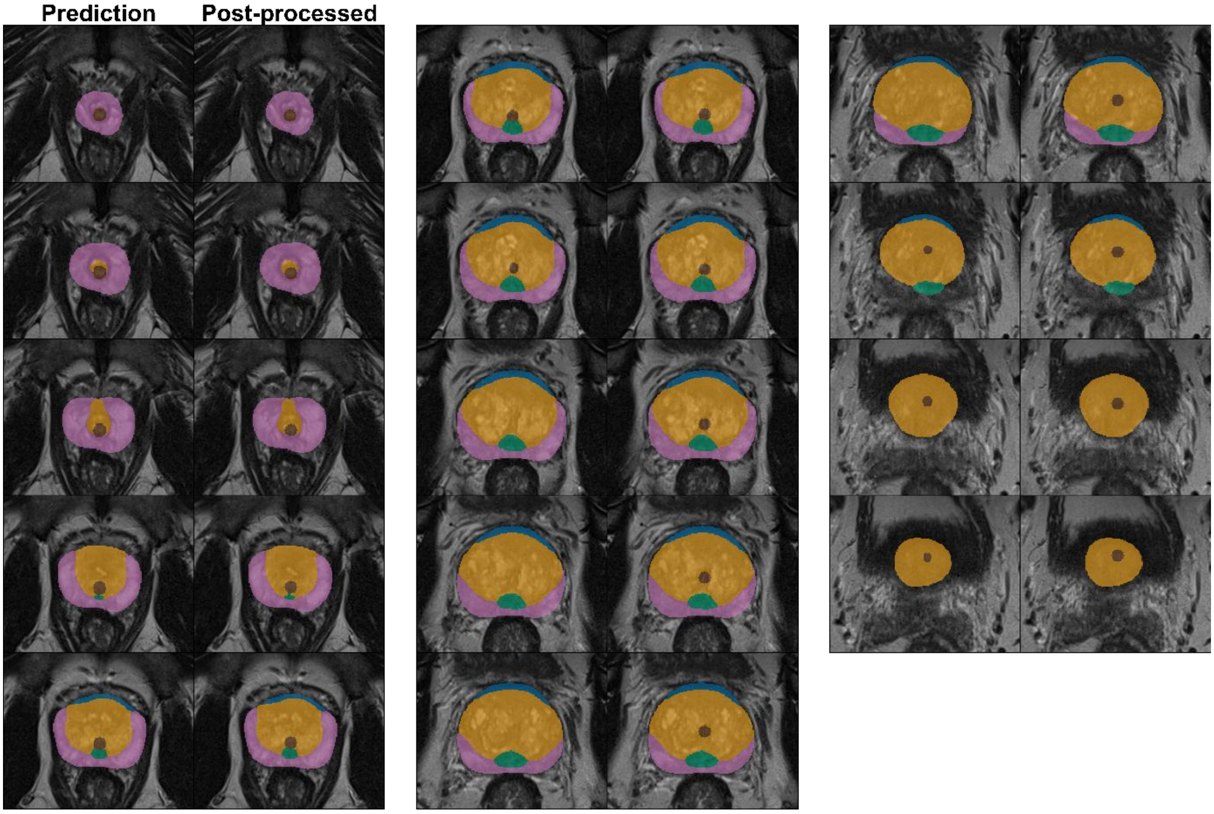

Figure S1: **Example prediction with and without post-processing.** An example where the post-processing (right column) assists with both adherence to the structure definition (circular, 6 mm diameter) and continuity of the urethra, compared to the original model prediction (left column).

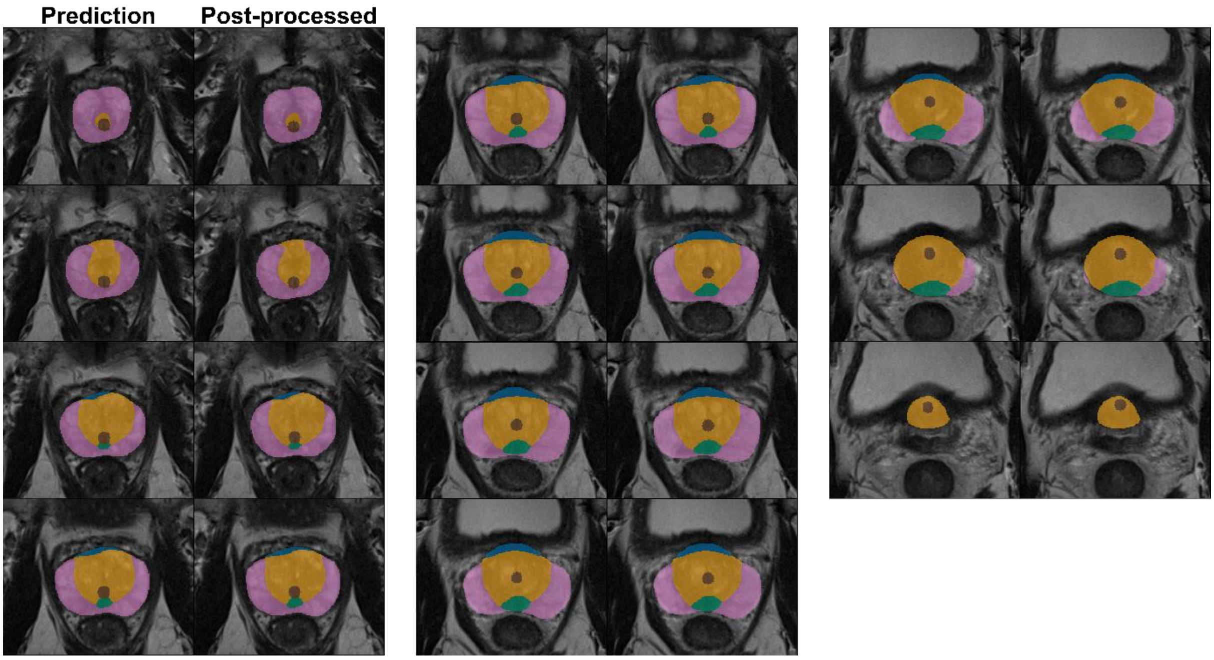

Figure S2: **Example prediction with and without post-processing.** An example where the post-processing (right column) assists with adherence to the structure definition (circular, 6 mm diameter) of the urethra, compared to the original model prediction (left column).

Table S1: **Impact of post-processing.** A quantitative comparison of the impact of the post-processing for the test set. Values are presented for each metric as the post-processed output subtracted by the prediction. All values represent the largest for any of the readers, and the smallest p-value.

Zone	Metric	Max Δ	Mean Δ	Median Δ	p-value
**Prostate**	DSC	0.0000	0.0000	0.0000	1.0
	SDSC *1 mm*	0.0000	0.0000	0.0000	1.0
**PZ**	DSC	-0.0026	-0.0004	-0.0004	<0.001
	SDSC *1 mm*	-0.0048	-0.0002	0.0002	0.335
**CZ**	DSC	-0.0132	-0.0009	-0.0003	0.192
	SDSC *1 mm*	0.0117	0.0008	0.0002	0.148
**TZ**	DSC	-0.0091	-0.0029	-0.0034	<0.001
	SDSC *1 mm*	0.0309	0.0044	0.0052	0.032
**AFS**	DSC	0.0014	0.0000	0.0000	0.180
	SDSC *1 mm*	0.0041	0.0001	0.0000	0.180
**Urethra**	DSC	0.1848	-0.0091	-0.0100	0.211
	SDSC *1 mm*	0.2049	0.0153	0.0072	0.282
	CLD	-9.4707	-0.7134	-0.0563	0.085

DSC: Dice Similarity Coefficient; SDSC: Surface DSC; CLD: Center Line Distance; Max/Mean/Median Δ: Difference between post-processed results relative to original prediction.

Analysis of volumes and zonal proportions

Table S2: **Comparison of prostate volumes.** A comparison of the prostate volumes in cm^3 in both test sets. For the ProstateZones test set each sample has been averaged between the two radiologists.

Dataset	n	median	IQR	mean	std	GMR (95% CI)	p-value
**ProstateZones**	40	51.8	39.1	58.5	26.6	1.41 (1.18 – 1.69)	<0.001
**External**	55	34.5	12.4	40.8	20.1	-	-

IQR: Inter Quartile Range; std: Standard Deviation; GMR: Geometric Mean Ratio; 95% CI: 95% Confidence Interval.

Table S3: **Comparison of zonal proportions.** A comparison of the zonal proportions relative to the prostate volume for each test set.

Structure	ProstateZones	External	OR (95% CI)	p-value
**PZ**	32.9%	36.4%	0.85 (0.69 – 1.05)	0.124
**CZ**	5.3%	10.8%	0.48 (0.40 – 0.58)	<0.001
**TZ**	56.6%	47.7%	1.45 (1.16 – 1.81)	0.001
**AFS**	2.9%	2.0%	1.58 (1.29 – 1.95)	<0.001
**Urethra**	2.3%	3.1%	0.72 (0.63 – 0.83)	<0.001

OR: Odds Ratio; 95% CI: 95% Confidence Interval.

Analysis of relation between CLD and structure volumes

Table S4: **Relationship between urethra displacement and volumes.** A comparison of the relationship between the CLD of the urethra and volumes of the prostate and prostate zones for both test sets. The Δ represents the CLD increase in mm per doubling of the structure volume.

Structure	Dataset	Pearson r	Spearman rho	Slope_log10	Δ (*mm*)
**Prostate**	ProstateZones	0.53	0.58	2.46	0.74
	External	0.44	0.41	3.40	1.02
**PZ**	ProstateZones	0.21	0.26	1.31	0.39
	External	0.02	-0.09	0.19	0.06
**CZ**	ProstateZones	0.41	0.39	2.34	0.70
	External	-0.15	-0.18	-1.08	-0.33
**TZ**	ProstateZones	0.56	0.65	1.90	0.57
	External	0.54	0.60	2.72	0.82
**AFS**	ProstateZones	0.05	0.06	0.24	0.07
	External	0.18	0.23	0.95	0.29

CLD: Center Line Distance; Δ: CLD increase in mm per doubling of volume.
